
JOURNAL INFORMATION
==============================
NLM Title Abbreviation: Medizinrecht
Journal ID: Medizinrecht
NLM Journal ID: 8309354

Medizinrecht

ISSN: 0723-8886
EISSN: 1433-8629

ARTICLE INFORMATION
==============================
PMCID: PMC8918894
PMID: 35310873
DOI: 10.1007/s00350-022-6136-7
Article ID: 6136
Article version: 1
Subjects: Aufsätze

Einwilligung und gesetzliche Forschungsklausel als Rechtsgrundlagen für die Sekundärnutzung klinischer Daten zu Forschungszwecken

Spitz Markus 1
Cornelius Kai 2
1 Rittershaus Rechtsanwälte, Mannheim, Deutschland
2 grid.9026.d 0000 0001 2287 2617 Fakultät für Rechtswissenschaft, Universität Hamburg, Hamburg, Deutschland
Electronic publication date: 2022 Mar 14
Print publication date: 2022
Volume: 40
Issue: 3
First page: 191
Last page: 198
Copyright: © Der/die Autor(en) 2022
License: Open Access Dieser Artikel wird unter der Creative Commons Namensnennung 4.0 International Lizenz veröffentlicht, welche die Nutzung, Vervielfältigung, Bearbeitung, Verbreitung und Wiedergabe in jeglichem Medium und Format erlaubt, sofern Sie den/die ursprünglichen Autor(en) und die Quelle ordnungsgemäß nennen, einen Link zur Creative Commons Lizenz beifügen und angeben, ob Änderungen vorgenommen wurden.
Die in diesem Artikel enthaltenen Bilder und sonstiges Drittmaterial unterliegen ebenfalls der genannten Creative Commons Lizenz, sofern sich aus der Abbildungslegende nichts anderes ergibt. Sofern das betreffende Material nicht unter der genannten Creative Commons Lizenz steht und die betreffende Handlung nicht nach gesetzlichen Vorschriften erlaubt ist, ist für die oben aufgeführten Weiterverwendungen des Materials die Einwilligung des jeweiligen Rechteinhabers einzuholen.
Weitere Details zur Lizenz entnehmen Sie bitte der Lizenzinformation auf http://creativecommons.org/licenses/by/4.0/deed.de.
License URL: https://creativecommons.org/licenses/by/4.0/

issue-copyright-statement: © Springer-Verlag GmbH Deutschland, ein Teil von Springer Nature 2022

==============================

**Markus Spitz und Kai Cornelius**

### A. Einleitung

Die Nutzung des Erkenntnispotenzials digitalisierter klinischer Daten steht nicht erst seit Ausbruch der weltweiten COVID-19-Pandemie auf der politischen Agenda. Bereits 2017 stellten die Gesundheitsminister der OECD-Staaten in einem gemeinsamen Statement fest *„[…] that access to, and the processing of, personal health data can serve health related public interests and bring significant benefits to individuals and society […].“*[1] Normativ treten bei der Weiternutzung klinischer Daten für Forschungszwecke unter anderem Fragen der Zweckbindung und -festlegung i. R. d. Einwilligung auf. Dies gilt insbesondere, wenn Daten aus dem Behandlungskontext für noch nicht konkret festgelegte Forschungszwecke gespeichert und nachfolgend längerfristig genutzt werden sollen. Dies ist etwa der Fall in dem dezentralen Speicherkonzept der vom BMBF geförderten Medizininformatik-Initiative (MI-I). Hier werden Daten aus der Krankenversorgung dezentral am Ort ihrer Entstehung gehalten und via vernetzter Datenintegrationszentren der beteiligten Konsortien, zu denen sich Universitätskliniken und weitere Partner zusammengeschlossen haben, standortübergreifend für viele verschiedene medizinische Forschungszwecke genutzt[2].

Solche umfangreichen und langfristigen Forschungsinfrastrukturen stellen den Gold-Standard informierter Einwilligung vor Herausforderungen. Konstruktiv werden die konkreten Forschungsfragen und -projekte sowie die nutzenden Forscher bei solchen Infrastrukturen erst im Lauf der Zeit konkretisiert. Dies wirkt sich auf die Möglichkeit einer detaillierten Aufklärung und Einwilligung aus. Insoweit stellen sich Fragen nach den Möglichkeiten von Einwilligungen mit weit gefasster Zweckbestimmung (*broad consent*). Deren Zulässigkeit wurde bislang speziell im Biobankkontext diskutiert, wo menschliche Körpersubstanzen bevorratend gelagert und nachfolgend für zu Beginn noch nicht detailliert festgelegte Forschungszwecke genutzt werden[3]. Ob und unter welchen Umständen ein solcher *broad consent* als Grundlage der Weiterverarbeitung klinischer Daten für Forschungszwecke nach geltendem Recht zulässig ist und welche weiteren Konzepte denkbar sind, um einen breiten wissenschaftlichen Nutzen klinischer Daten mit einem angemessenen Betroffenenschutz zu vereinbaren, soll nachfolgend untersucht werden.

### B. Sekundäre Forschungsnutzung als Weiterverarbeitung personenbezogener Daten

Der Zweck, für den die vorliegend betrachteten klinischen Daten erhoben wurden, liegt insbesondere in der ärztlichen Dokumentationspflicht (§ 630f BGB, § 10 MBO-Ä) sowie der sozialrechtlichen Leistungsabrechnung als Teil der Patientenbehandlung[4]. Jedes Speichern, Übermitteln oder sonstige Verarbeiten der klinischen Daten, das nicht der individuellen Patientenbehandlung, sondern überindividuellen Forschungszwecken dient, stellt eine zweckändernde, rechtfertigungsbedürftige Weiterverarbeitung personenbezogener Daten dar (Art. 5 Abs. 1 lit. a und b DSGVO)[5].

Nach Art. 5 Abs. 1 lit. b 2. Halbs. DSGVO gilt eine Weiterverarbeitung für Forschungszwecke unter Beachtung des Art. 89 Abs. 1 DSGVO nicht als mit dem ursprünglichen Zweck unvereinbar. Teilweise wird die Auffassung vertreten, aus der fingierten Zweckkompatibilität folge i. V. mit Erwägungsgrund 50 S. 2 DSGVO die Möglichkeit, die Weiterverarbeitung für Forschungszwecke auf die Rechtsgrundlage der Datenerhebung zu stützen[6]. Die Auffassung würde indes speziell bei der Verarbeitung besonderer Kategorien von Daten, die zusätzlich zum allgemeinen Erlaubnisvorbehalt nur unter den verschärften Bedingungen des Art. 9 Abs. 2 DSGVO zulässig ist, zu Systembrüchen führen. Es ist zweifelhaft, dass der europäische Gesetzgeber die Schaffung eines zusätzlichen Erlaubnistatbestandes (in Gestalt der Grundlage der Ersterhebung) für zweckkompatible Forschungszwecke intendierte und dies ausschließlich in dem (nicht verbindlichen und nur zur Auslegung heranzuziehenden) Erwägungsgrund (!) 50 S. 2 DSGVO[7] zum Ausdruck gebracht haben soll[8]. Rechtsfolge des Art. 5 Abs. 1

Rechtsanwalt Markus Spitz, Im Projekt LinCDat wissenschaftlicher Mitarbeiter an der Universität Heidelberg, nunmehr tätig bei Rittershaus Rechtsanwälte, Mannheim, Deutschland

Prof. Dr. iur. Kai Cornelius, Fakultät für Rechtswissenschaft, Universität Hamburg, Deutschland

*) Dieser Beitrag entstand im Rahmen des Forschungsprojekts Learning from Clinical Data (LinCDat): Sekundärnutzung klinischer Daten für Forschung und Versorgung – ethische, gesellschaftliche und rechtliche Aspekte, das von der Deutschen Forschungsgemeinschaft (DFG) gefördert wird (Projektnummer: 406103282). Die Verfasser danken ihren Projektmitgliedern Frau Prof. Dr. *Eva Winkler*, Frau Dr. *Katja Mehlis*, Frau *Anja Köngeter*, Herrn Dr. *Christoph Schickhardt*, Herrn Dr. *Martin Jungkunz* und Herrn *Adrian Thorogood* LL.M. für ihre wertvolle Unterstützung.

1) *OECD Health Ministerial Statement*, 2017, S. 7, https://www.oecd.org/health/ministerial/ministerial-statement-2017.pdf (8. 2. 2022).

2) MI-I, Konsortien, https://www.medizininformatik-initiative.de/de/konsortien (8. 2. 2022).

3) *Augsberg/v. Ulmenstein*, GesR 2018, 341, 345; *Koch*, Das Biobankgeheimnis, 2013, S. 171 ff. m. w. N. Zum „Doppelcharakter“ von Biobanken als Proben- und Datenbanken vgl. *Albers*, MedR 2013, 483; *Koch*, in: *Gropp et al.*, GS f. *Heine*, 2016, S. 200; *Taupitz*, in: *Katzenmeier*, FS f. *Hart*, 2020, S. 603.

4) Hierzu ausführlich *Jungkunz et al.*, in: *Richter et al.*, Datenreiche Medizin, 2021, 185 ff.

5) Dies gilt auch dann, wenn die datenerhebende Stelle die klinischen Daten zuvor pseudonymisiert oder verschlüsselt hat (Art. 4 Nr. 5 DSGVO), bevor sie sie für Forschungszwecke zur Verfügung stellt. Die Übermittlung pseudonymisierter Daten bleibt rechtfertigungsbedürftige Datenverarbeitung für die Stelle, die weiterhin Zugriff auf den Zuordnungsschlüssel hat und den Betroffenen re-identifizieren kann, *Klar/Kühling*, in: *Kühling/Buchner*, DSGVO, 3. Aufl. 2020, Art. 4 Nr. 5, Rdnr. 11; *Roßnagel/Scholz*, MMR 2000, 721, 730; *Scholz*, in: *Simitis*, BDSG a. F., 8. Aufl. 2014, § 3, Rdnr. 219b; a. A. *v. Kielmansegg*, in: TMF-Stellungnahme „Datenspende“, 2020, S. 91; *Schneider*, Sekundärnutzung klinischer Daten, 2015, S. 24.

6) Vorliegend z. B. die Einwilligung in die Behandlung oder den Behandlungsvertrag. In diese Richtung etwa *Golla*, in: *Mantz et al.*, Handbuch Datenschutz, 2019, § 23, Rdnr. 56.

7) Erwägungsgründe, die auch im verfügenden Teil einen Anhalt finden, sind bei der Auslegung zu berücksichtigen, vgl. *Riesenhuber*, in: *ders.*, Europäische Methodenlehre, 4. Aufl. 2021, § 10, Rdnr. 38.

8) *Herbst*, in: *Kühling/Buchner*, DSGVO, 3. Aufl. 2020, Art. 5, Rdnr. 48.

lit. b 2. Halbs. DSGVO ist vielmehr, dass der weiterverarbeitende Forschungszweck unter den Voraussetzungen des Art. 89 Abs. 1 DSGVO als zweckkompatibel gilt und keines Kompatibilitätstests (Art. 6 Abs. 4 DSGVO) bedarf[9]. Daneben muss die Weiterverarbeitung für Forschungszwecke, wie stets, durch Einwilligung oder eine andere legitime Grundlage gerechtfertigt werden.

### C. Rechtsgrundlage Einwilligung

Als Rechtfertigung kommt die informierte Einwilligung in Betracht. Hierbei handelt es sich um die freiwillige, in informierter Weise, für den bestimmten Fall abgegebene Willensbekundung des einsichts- und urteilsfähigen Betroffenen (Art. 4 Nr. 11 DSGVO).

#### *I. Aufklärungs- und Legitimationsumfang*

Der Detailgrad der informierten Weise und des bestimmten Falls können im Forschungskontext in einem Spannungsfeld zur offenen Vorgehensweise der Forschung stehen. Dies führt zur eingangs aufgeworfenen Frage nach der Möglichkeit einer weiten Zweckfestsetzung i. R. d. Einwilligung.

##### 1. Praxisbeispiel

Einen *broad consent* als breite Fassung des inhaltlichen Verarbeitungszwecks sowie der institutionell nutzungsberechtigten Forschungsstellen sieht der Mustereinwilligungstext der MI-I vor[10]. Hiernach sollen „Patientendaten […] im Sinne eines breiten Nutzens für die Allgemeinheit für viele verschiedene Forschungszwecke verwendet werden“. Es wird darauf hingewiesen, dass „zum jetzigen Zeitpunkt […] noch nicht alle zukünftigen medizinischen Forschungsinhalte beschrieben werden“ können, sondern diese sich „sowohl auf ganze Krankheitsgebiete (z. B. Krebsleiden, Herz-Kreislauf-Erkrankungen, Erkrankungen des Gehirns) als auch auf heute zum Teil noch unbekannte einzelne Krankheiten […] beziehen“ können. Die Wirkung der Einwilligung für die Nutzung der Patientendaten ist zeitlich auf 30 Jahre befristet, wobei die Einwilligung auch für Behandlungsdaten genutzt werden kann, die innerhalb von fünf Jahren nach der Einwilligungserteilung neu hinzukommen.

Bei der institutionellen Zweckfestlegung sieht die Mustereinwilligung die wissenschaftliche Analyse und Nutzung der codierten (pseudonymisierten) Patientendaten[11] durch Dritte „wie z. B. durch andere Universitäten/Institute/forschende Unternehmen“ vor. Zugriff auf die reidentifizierende Zeichenkombination hat ausschließlich eine unabhängige interne Stelle oder Datentreuhandstelle. Jedes Forschungsvorhaben bedarf zudem der zustimmenden Bewertung durch eine Ethik-Kommission.

##### 2. Auslegung der Zweckfestlegung i. R. d. Einwilligung

In dem genannten Mustertext wird die legitimierende Einwilligung für einen sowohl sachlich (Forschungszweck, Forschungsfrage, zu beforschende Krankheit) als auch institutionell (datenempfangende und datennutzende Forschungsstelle) generischen, nicht konkret festgelegten Verarbeitungszweck eingeholt und hierüber entsprechend aufgeklärt. Ob dies den normativen Anforderungen entspricht, muss durch Auslegung der einschlägigen DSGVO-Regeln ermittelt werden[12].

###### a) Wortlaut

Bei der Wortlautauslegung gilt es zunächst zu beachten, dass insoweit alle 24 Sprachfassungen gleich autoritativ und bei der Auslegung von Unionsrecht zu beachten sind[13]. Nach der deutschen Fassung des Art. 4 Nr. 11 DSGVO muss die Einwilligung in „informierter Weise“[14] für einen „bestimmten Fall“[15] erteilt werden. Der bestimmte Fall gibt eine gewisse Fall- und Zweckbegrenzung vor. Wo die Grenze verläuft und ob auch die bloße Angabe einer wissenschaftlichen Disziplin (etwa biomedizinische Forschung) genügt, bleibt zunächst offen.

Präziser ist der Wortlaut in Art. 6 Abs. 1 lit. a DSGVO und Art. 9 Abs. 2 lit. a DSGVO. Nach Art. 6 Abs. 1 lit. a DSGVO muss die Einwilligung zur Verarbeitung der betreffenden Daten „für einen oder mehrere bestimmte Zwecke gegeben“ werden[16]. Art. 9 Abs. 2 lit. a DSGVO verlangt für besondere Kategorien von Daten, dass die Einwilligung für „festgelegte Zwecke“ eingeholt wird[17]. Dies deutet auf ein in Art. 9 DSGVO gegenüber Art. 6 DSGVO gesteigertes Maß an Bestimmtheit der Zweckfestlegung hin[18]. Der Vergleich der Sprachfassungen zeigt, dass der festgelegte Zweck einen spezifizierten Zweck meint[19].

Nach dem Wortlaut kann die Einwilligung zwar gleichzeitig für eine Vielzahl an Verarbeitungszwecken erklärt werden („mehrere Zwecke“). Andererseits müssen alle diese Zwecke festgelegt (spezifiziert) sein. Ob dies bei der Zweckfestlegung „für sämtliche Forschungszwecke“ oder auch „für medizinische Forschung“ der Fall ist, scheint zweifelhaft[20]. Jedenfalls in einer Zweckbeschreibung „für sämtliche Forschungszwecke“ ohne fachliche Präzision liegt keine adäquate Konkretisierung. Auch die weite Vorhabenfestlegung „für medizinische Forschung“ erlaubt eine Vielzahl denkbarer Verarbeitungskonstellation und bezieht auch Teilbereiche ein, die gesellschaftlich und individuell kritisch bewertet werden könnten, etwa medizinisch-militärische Forschung, Human Enhancement oder Forschung mit kommerziellem Nutzen[21].

###### b) Systematik

Systematisch ist die Interdependenz zwischen Zweckfestlegung (Art. 5 Abs. 1 lit. b DSGVO) und Einwilligung (Art. 6 Abs. 1 lit. a und Art. 9 Abs. 2 lit. a DSGVO) zu sehen[22]. Die Zweckfestlegung versichert dem Einwilligenden, dass alle Verarbeitungsvorgänge nur dem(n) bewilligten Zweck(en) dient(en) (Erwägungsgrund 32 S. 4 DSGVO)[23]. Zweckfestlegung und Zweckbindung sind daher sowohl auf grundrechtlicher Ebene (Art. 8 Abs. 2 GRCh) als auch i. R. der Betrof-

9) *Albrecht/Jotzo*, Datenschutzrecht, 2017, Teil 3, Rdnrn. 55 f.; *Weichert*, ZD 2020, 18, 21; *Werkmeister/Schwaab*, CR 2019, 85, 88.
10) MI-I, Mustertext Patienteneinwilligung V 1.6d, 2020, S. 1 ff.; eine ähnlich gestaltete breite Einwilligung enthält der Mustertext des Arbeitskreises Medizinischer Ethik-Kommission (AKEK), der insoweit mit Varianten arbeitet, AKEK, Mustertext V 3.1, 2019, Pkt 3 und 7, S. 3 ff.
11) Der Schlüssel soll von einer unabhängigen externen Treuhandstelle verwaltet werden.
12) Die DSGVO ist autonom vom Recht der Mitgliedstaaten auszulegen. Die Auslegungsmethoden sind mit denen der nationalen Rechtsordnungen vergleichbar, *Riesenhuber*, in: *ders.*, Europäische Methodenlehre, 4. Aufl. 2021, § 10, Rdnr. 12.
13) *Cornelius*, NZWiSt 2016, 421, 424; *Riesenhuber*, in: *ders.*, Europäische Methodenlehre, 4. Aufl. 2021, § 10, Rdnr. 14.
14) Engl: „specific“, Franz: „spécifique“.
15) Engl: „informed“, Franz: „éclairée“.
16) Engl: „one or more specific purposes“, Franz: „une ou plusieurs finalités spécifiques“.
17) Engl: „one or more specified purposes“, Franz: „une ou plusieurs finalités spécifiques“.
18) Zumindest in der deutschen und engl Fassung.
19) *Petri*, in: *Simitis et al.*, Datenschutzrecht, 2019, Art. 9, Rdnr. 32.
20) *Spranger*, MedR 2017, 864; anders *Schaar*, ZD 2017, 213, 215, die hieraus die breite Einwilligung folgert.
21) Der Mustertext der MI-I, Patienteneinwilligung, V 1.6d, 2020, S. 1 sieht entsprechend einen Negativausschluss vor, wonach die Patientendaten ausschließlich für medizinische Forschung genutzt werden, die der verbesserten Erkennung, Behandlung und Vorbeugung von Krankheiten dient und nicht für die Entwicklung biologischer Waffen oder für diskriminierende Forschungsziele.
22) Das BVerfG, NJW 2020, 300, 307 spricht von der Wechselwirkung von Zweckbindung und Einwilligungserfordernis.
23) Vgl. *Simitis*, MedR 1985, 195, 198.

fenenrechte und Verantwortlichenpflichten[24] relevant. Eine außerhalb des festgelegten Zwecks erfolgende Datenverarbeitung muss als Zweckänderung mit dem ursprünglichen Zweck kompatibel und legitimiert sein. Diese autonomiesichernde Kontrollfunktion spricht dafür, dass die Betroffenen so präzise wie möglich über den Verarbeitungszweck informiert werden müssen[25]. Je weiter und unpräziser die Zweckfestlegung ausfällt, desto weiter und unvorhersehbarer wird der Verarbeitungsumfang, der auf die Einwilligung gestützt werden kann[26]. Dadurch droht die mit dem bewilligten Zweck verbundene Verarbeitungsbeschränkung ausgehöhlt zu werden. Zwar sieht Art. 5 Abs. 1 lit. b 2. Var. DSGVO speziell im Forschungskontext Lockerungen des Zweckbindungsgrundsatzes vor. Diese befreien jedoch nur vom Kompatibilitätstest nach Art. 6 Abs. 4 DSGVO und lassen den Legitimationsumfang der zusätzlich erforderlichen Rechtsgrundlage unberührt[27]. Der autonomiesichernde systematische Zusammenhang von Einwilligung und Zweckbindung spricht dafür, dass Betroffene wenigstens die Wahl haben müssen, ob sie in einen konkreten oder weiten Zweck einwilligen wollen.

c) Entstehungsgeschichte

Die Frage nach der Möglichkeit einer breiten Zweckfestlegung war schon vor Erlass der DSGVO umstritten. Deshalb wurde auf Betreiben der deutschen Delegation[28] in Erwägungsgrund 33 DSGVO (ex Erwägungsgrund 25) aufgenommen, dass es Betroffenen erlaubt sein solle, „ihre Einwilligung für bestimmte Bereiche wissenschaftlicher Forschung zu geben, wenn dies unter Einhaltung der anerkannten ethischen Standards für die wissenschaftliche Forschung geschieht". Aus Erwägungsgrund 33 DSGVO wird nicht selten der gesetzgeberische Wille zur Regelung einer breiten Einwilligung für wissenschaftliche Forschung abgeleitet[29]. Gleichwohl deutet die Formulierung in Erwägungsgrund 33 S. 1 und 2 DSGVO darauf hin, dass die Einwilligung in den weiten Forschungszweck nur ausnahmsweise („*Oftmals* kann der […] wissenschaftlichen Forschung zum Zeitpunkt der Erhebung […] nicht vollständig angegeben werden" – Herv. *d. Verf.*) und nur nach Wahl der Betroffenen („sollte es betroffenen Personen erlaubt sein") erfolgen soll. Für den vom europäischen Gesetzgeber intendierten ausnahmsweisen Charakter spricht zudem die Tatsache, dass die Zweckerweiterung erst in der Spätphase der Trilog-Verhandlungen Eingang in die Erwägungsgründe gefunden hat. Der eigentliche Normtext geht i. R. d. Rechtsgrundlage ausnahmslos von einem festgelegten und spezifischen Zweck aus[30].

d) Telos

Bei der Auslegung von Unionsrecht kommt der teleologischen Auslegung gesteigerte Bedeutung zu[31]. Dies wird vorliegend durch die Grundrechtssensibilität der Verarbeitung von Gesundheitsdaten zu Forschungszwecken noch verstärkt. Im interventionellen Behandlungs- und Forschungskontext sichert die Einwilligung als fundamentales Prinzip die Autonomie und Würde des Einzelnen zur Selbstbestimmung über den Umgang mit seinem Körper[32]. Auch bei der Weiterverarbeitung der hierbei gewonnenen klinischen Daten kommt der informierten Patienteneinwilligung eine wichtige Rolle zu[33]. Sie ist genuiner Ausdruck informationeller[34] und medizinischer[35] Selbstbestimmung. Sie zielt auf die Befähigung der Patienten zur Bestimmung über den Umgang mit den sie betreffenden Daten im Anbetracht möglicher Gefahren und Risiken und letztlich zur informierten Partizipation, in Abkehr von einem paternalistischen Umgang mit Patienten und Patientendaten ab[36]. Sowohl die Therapie als auch der Datenumgang stützen sich in der modernen Medizin auf einen durch Vertrauen geprägten Informationsfluss und von Patienten verstandene und konsentierte Verfahren[37].

Die autonomiesichernde Funktion informierter Einwilligung bedingt, dass sich der durch sie legitimierte Verarbeitungsvorgang und -zweck im Rahmen der erteilten Einwilligung bewegt[38]. Um dies zu gewährleisten, muss der Legitimationsumfang der Einwilligung nach Inhalt, Zweck und Tragweite hinreichend konkretisiert den Betroffenen vermittelt werden, um diese in die Lage einer echten Entscheidung zu versetzen[39]. Dies gilt besonders im Gesundheitskontext, für den Art. 9 Abs. 2 lit. a DSGVO entsprechend gesteigerte Anforderungen bereithält.

Es erscheint fraglich, ob die Einwilligung in einen nur breit festgelegten Forschungszweck, ausgeführt von nicht konkret festgelegten Forschungsakteuren, diesen Anforderungen genügt. Dies dürfte zumindest zu verneinen sein, wenn der Rückgriff auf die breite Zweckfestlegung als Teil der informierten Einwilligung den Regelfall gerade in groß angelegten Forschungsprojekten bildet. Hierfür spricht auch, dass die Frage nach der Bestimmtheit der erforderlichen Aufklärung regelmäßig nicht pauschal, sondern nur im Einzelfall anhand der Tragweite für die Rechte und Freiheiten der Betroffenen bestimmt werden kann[40]. Diese Tragweite ist in Anbetracht der sensiblen medizinischen Daten als tendenziell erheblich einzustufen. Zwar müssen die Betroffenen nicht über jedes Detail der Datennutzung informiert werden. Jedoch sind solche Informationen notwendig, die für ihre Entscheidung relevant sind[41]. Ein zu kleinteiliger Informations- und Einwilligungsprozess birgt die Gefahr, dass die Betroffenen vom *information overload* überfordert werden[42]. Die Patienten müssen jedoch zumindest aus ihrer Laienosphäre Kenntnis von Zweck, Eigenart, Bedeutung und Tragweite der Weiterverarbeitung sowie den forschenden Stellen haben, um ihre Einwilligungsentscheidung auf eine freie und eigenständige Nutzen-Risiko-

24) Informationspflicht über den Verarbeitungszweck (Art. 13 und 14 je Abs. 1 lit. c DSGVO), Löschpflicht bei Zweckerreichung (Art. 17 Abs. 1 lit. a DSGVO) oder Pflicht zweckkompatibler Weiterverarbeitung (Art. 5 Abs. 1 lit. b, 6 Abs. 4 DSGVO).
25) *Buchner/Kühling*, in: *Kühling/Buchner*, DSGVO, 3. Aufl. 2020, Art. 7, Rdnr. 61; *Pöttgen*, Medizinische Forschung und Datenschutz, 2009, S. 107 f.
26) *Weichert*, ZD 2020, 18, 21.
27) S. oben B.
28) German delegation to Working Party, 4. 2. 2015, Subject Consent, 14707/2/14 REV 2, S. 3, https://data.consilium.europa.eu/doc/document/ST-14707-2014-REV-2/en/pdf (8. 2. 2022).
29) *Buchner/Kühling*, in: *Kühling/Buchner*, DSGVO, 3. Aufl. 2020, Art. 7, Rdnr. 64; *Rammos*, DSRITB 2017, 359, 368; *Schantz/Wolff*, Das neue Datenschutzrecht, 2017, Rdnr. 527; allgemein zur Ermittlung des Gesetzgeberwillens aus Erwägungsgründen *Riesenhuber*, in: *ders.*, Europäische Methodenlehre, 4. Aufl. 2021, § 10, Rdnr. 38.
30) Entsprechend führt der *EDPS*, Opinion on scientific research, 2020, S. 19 aus: „Recital 33 does not however take precedence over the conditions for consent set out in Articles 4(11), 6(1)(a), 7 and 9(2)(a) of the GDPR".
31) *Wegener*, in: *Calliess/Ruffert*, EUV/AEUV, 6. Aufl. 2022, Art. 19 EUV, Rdnr. 32.
32) *Allen/McNamara*, Bioethics 25 (2011), 155, 156; vgl. auch die Deklaration von Helsinki.
33) EDPS, Opinion on data protection and scientific research, 2020, S. 19.
34) *Roßnagel/Pfitzmann/Garstka*, DuD 2001, 253, 258.
35) BVerfGE 52, 131, 167.
36) Zu informierten und paternalistischen Modellen in der Arzt-Patienten-Beziehung *Emanuel/Emanuel*, JAMA 1992, 2221 ff.
37) *Simitis*, in: *Abt et al.*, Krankendaten, 1985, S. 56, 57.
38) *Schild*, in: *Wolff/Brink*, BeckOK DSGVO, 38. Ed 2021, Art. 4, Rdnr. 125.
39) *Radlanski*, Das Konzept der Einwilligung, 2015, S. 71.
40) *Jandt/Hornung/Roßnagel*, Datenschutz in der medizinischen Forschung, 2009, C14, S. 13.
41) *Pommerening et al.*, Datenschutz in medizinischen Forschungsprojekten, 2014, S. 39.
42) Zum *information overload* als Funktionsdefizit der informierten Einwilligung vgl. *Dorneck et al.*, MedR 2019, 431, 433 f.; als Desinformationsmittel vgl. *Ingold*, Desinformationsrecht, 2011, S. 33.

Abwägung stützen zu können[43]. Ein solches grundlegendes Verständnis ist erforderlich, damit die Betroffenen ihre Kontrollrechte adäquat ausüben können[44].

Aus der Auslegung der einschlägigen Regelungen folgt, dass eine breite Einwilligung zwar unter Umständen möglich ist, dies aber auf Ausnahmen beschränkt bleiben muss. Den Regelfall muss auch im biomedizinischen Forschungskontext und gerade beim Umgang mit Gesundheitsdaten die bestimmte, informierte Einwilligung bilden.

#### 3. Sichtweise der europäischen und nationalen Aufsichtsbehörden

Entsprechend kritisch steht auch das European Data Protection Board (EDPB) der pauschalen Nutzung breiter Einwilligung i.R. medizinischer Forschung gegenüber[45]. Das EDPB führt aus, dass Erwägungsgrund 33 DSGVO zwar das Maß an Spezifizierung und Granularität der Einwilligung bei wissenschaftlicher Forschung etwas flexibler zu gestalten scheine. Jedoch folge bei besonderen Datenkategorien aus Art. 9 DSGVO eine strikte Auslegung des Erwägungsgrund 33 DSGVO. Deshalb müsse auch im Forschungskontext die Einwilligung regelmäßig auf einem gut beschriebenen Zweck beruhen, der nur ausnahmsweise allgemeiner ausfallen dürfe, wenn der Zweck am Anfang eines Forschungsprojekts nicht vollständig angegeben werden könne. In diesem Fall müsse der Verantwortliche andere Wege suchen, um der informierten Einwilligung zu entsprechen[46]. Diese anderen Wege könnten darin bestehen, den Betroffenen die Möglichkeit zu geben, in allgemein beschriebene Forschungszwecke sowie in spezielle Phasen des Forschungsprojekts, die anfangs schon bekannt sind, einzuwilligen (stufenweise Einwilligung). Mit Fortschreiten der Forschung solle die Einwilligung für später konkretisierbare Projektschritte eingeholt werden, bevor die nächste Phase beginne[47]. Auch im Falle eines solchen stufenweisen und dynamischen Vorgehens müsse sichergestellt werden, dass die Forschung den anzuwendenden ethischen Standards entspreche[48]. Hierbei könne auch das Vorhalten eines transparenten Forschungsplans helfen[49].

Auch die nationalen Aufsichtsbehörden sprechen sich für eine restriktive Handhabung der breiten Einwilligung aus. So äußert die Datenschutzkonferenz (DSK)[50], dass nach Art. 4 Nr. 11 DSGVO eine Einwilligung stets für den bestimmten Fall abzugeben sei[51]. Das Erfordernis des bestimmten Falls konkretisiere den Zweckbindungsgrundsatz, wonach personenbezogene Daten für festgelegte, eindeutige und legitime Zwecke zu erheben seien. Nur wenn das konkrete Design des Forschungsvorhabens eine vollständige Zweckbestimmung im Vorfeld der Verarbeitung nicht zulasse, könne eine breite Einwilligung genutzt werden. Gleichwohl entbinde Erwägungsgrund 33 DSGVO nicht von der Pflicht, eingrenzende Mechanismen vorzusehen. Als Korrektiv sei insbesondere zu prüfen, „ob das Arbeiten mit einem dynamic consent möglich" sei bzw. eine „Widerspruchsmöglichkeit" des Betroffenen vorliege[52].

Dem folgt auch der Bundesbeauftragte für den Datenschutz und die Informationsfreiheit (BfDI). In seiner Stellungnahme zu §363 Abs. 8 SGB V, der wie §67b Abs. 3 S. 1 SGB X auf Ebene des Sozialdatenschutzrechts bei der Verarbeitung von Sozialdaten eine Einwilligung für bestimmte Bereiche wissenschaftlicher Forschung zulässt, merkt der BfDI kritisch an, dass die Regelung eines *broad consent* als Rechtsgrundlage nur unter bestimmten Voraussetzungen und unter Einhaltung zusätzlicher Sicherungsmaßnahmen statthaft sei: „Nur wenn das konkrete Design des Forschungsvorhabens eine vollständige Zweckbestimmung nicht zulässt, können […] Abstriche hinsichtlich der Bestimmtheit des Zwecks hingenommen werden. Dann sind allerdings zusätzliche Sicherungsmaßnahmen zu ergreifen."[53] Auch die Gesetzesbegründung zu §67b Abs. 3 SGB X konzediert, dass im Fall offener Zweckfestlegung dem Grundsatz informierter Einwilligung Rechnung getragen und die Forschungsfrage schrittweise konkretisiert werden müsse[54].

### *II. Stellungnahme*

#### 1. Ausnahmecharakter breiter Einwilligung

Diesem Ausnahmeprinzip breiter Zweckfestlegung ist im Angesicht der dargestellten Datenschutzregeln zuzustimmen. Die breite Zweckfestlegung, die den Betroffenen sowohl bezogen auf den sachlichen Forschungszweck als auch die jeweils institutionell forschende Stelle generisch und allgemein aufklärt, hat sich auf den folgenden Ausnahmefall zu beschränken: Die vorherige Festlegung des Forschungszwecks ist aufgrund des inkrementellen und unvoreingenommenen wissenschaftlichen Erkenntnisstrebens schlechterdings unmöglich[55]. Entsprechend den Hinweisen des EDPB sind die Betroffenen jedoch im Verlauf eines Forschungsprojekts dynamisch aufzuklären und ihre Einwilligung konkret einzuholen, sobald dies möglich ist.

Hieraus ergibt sich gerade bei langfristigen Forschungsvorhaben ein differenziertes Einwilligungskonzept je nach Forschungsphase: Der Forschungszweck wird regelmäßig dort noch nicht konkret festgelegt werden können, wo klinische Daten für Forschungszwecke zunächst bevorratend gespeichert werden. Dies ist etwa in Forschungsdatenzentren der Fall. Dabei erfolgt bereits die Übermittlung und Speicherung der Daten für nachfolgende Forschungszwecke außerhalb des ursprünglichen Behandlungskontextes und bedarf der Rechtfertigung. Zu diesem frühen Zeitpunkt wird jedoch gerade bei groß angelegten Projekten kaum jemals abschließend festgelegt werden können, welchen Forschungszielen und Einrichtungen die Daten in der Folge dienen können. Hier besteht ein dringendes Bedürfnis für die Möglichkeit breiter Einwilligung zum Aufbau solcher Forschungsdatenbanken[56]. Das durch den weiten Zweck bei der Datenspeicherung in der Forschungsdatenbank verringerte Maß an Autonomie ist zu kompensieren, beispielsweise durch die Publikation von Nutzungsordnungen oder Forschungsplänen, die dem Betroffenen das Ziel und die Methoden der Datensammlung erläutern[57].

43) *Koch*, Das Biobankgeheimnis, 2013, S. 175; ähnlich für eine Interpretation des *broad consent* im Wege einer „schutzgutorientierte[n] Annäherung an das Konzept des „informed consent" unter Berücksichtigung des Wesensgehalts von Art. 8 GRCh" *Dochow*, MedR 2021, 115, 123.
44) EDPB, Leitlinien 5/2020, 4.5.2020, S. 38.
45) EDPB, Leitlinien 5/2020, 4.5.2020, S. 36ff., Rdnrn. 154ff., ähnlich die Art.-29-Gruppe, WP 259, 10.4.2018, S. 34ff.
46) EDPB, Leitlinien 5/2020, 4.5.2020, S. 37, Rdnrn. 156–158.
47) EDPB, Leitlinien 5/2020, 4.5.2020, S. 37, Rdnr. 158.
48) EDPB, Leitlinien 5/2020, 4.5.2020, S. 37, Rdnr. 158.
49) EDPB, Leitlinien 5/2020, 4.5.2020, S. 38, Rdnr. 161.
50) Die Datenschutzkonferenz ist das Gremium der unabhängigen deutschen Datenschutzaufsichtsbehörden des Bundes und der Länder.
51) DSK, Auslegung „bestimmte Bereiche wissenschaftlicher Forschung", 3.4.2019, S. 1ff.
52) DSK, Auslegung „bestimmte Bereiche wissenschaftlicher Forschung", 3.4.2019, S. 2.
53) BfDI, Stellungnahme zum PDSG v. 3.4.2020, S. 15; so schon in 27. TB 2017–2018, S. 57.
54) BT-Dr. 18/12611, S. 104.
55) Die Zulässigkeit einer solchen anfänglich generischen Angabe von Empfängerkategorien (im Beispiel der MI-I „Universitäten, Instituten oder forschenden Unternehmen") kann auch aus der Informationspflicht nach Art. 13 Abs. 1 lit. e DSGVO abgeleitet werden.
56) Von der Möglichkeit zum Aufbau von Forschungsrepositorien geht auch Erwägungsgrund 157 DSGVO aus.
57) Vgl. DSK, Auslegung „bestimmte Bereiche wissenschaftlicher Forschung", 3.4.2019, S. 2; zum Forschungsplan auch EDPB, Leitlinien 5/2020, 4.5.2020, S. 38, Rdnr. 161.

#### 2. Dynamische Einwilligung im Forschungsverlauf

Kann im zeitlichen Verlauf der Forschungszweck in sachlicher und institutioneller Hinsicht jedoch konkretisiert werden, entfällt die Rechtfertigung für die ausnahmsweise breite Zweckfestlegung. Analog zum dynamisch fortschreitenden Forschungsvorhaben muss auch die Einwilligung dynamisch für die konkretisierbaren Forschungszwecke fortschreiten (*dynamic consent*)[58]. Insbesondere müssen die Betroffenen erfahren, welche Institutionen die sie betreffenden klinischen Daten für Forschungszwecke nutzen[59]. Schließlich können sie ein nachvollziehbares Interesse daran haben, dass bestimmte forschende Unternehmen oder bestimmte im zeitlichen Verlauf auftretende Forschungsthemen nicht von der Nutzung der sie betreffenden Daten profitieren sollen.

Dies führt zu einem auf zeitlicher Achse dynamischen, repetitiven Aufklärungs- und Einwilligungsvorgang. Dabei muss die konkretisierte Einwilligung nicht bei jeder trivialen Änderung des Forschungsumgangs eingeholt werden. Die Notwendigkeit einer erneuten Einwilligung richtet sich danach, ob mit dem neuen Forschungsumgang eine Zweckänderung verbunden ist. Dies setzt eine wesentliche inhaltliche Änderung der Forschungsnutzung voraus, etwa ein neues Forschungsprojekt, eine wesentlich abweichende Forschungsfrage oder eine institutionelle oder organisatorische Änderung, etwa durch die Datennutzung einer anderen verantwortlichen Forschungseinrichtung. In jedem Fall muss es den Betroffenen überlassen werden, ob sie es bei der initialen breiten Einwilligung belassen wollen oder ob sie bei der Datennutzung für inhaltlich und institutionell völlig divergierende Forschungsprojekte erneut informiert werden und einwilligen möchten. Diese Wahlmöglichkeit gibt auch Erwägungsgrund 33 DSGVO vor[60]. Eine solche Wahlmöglichkeit fehlt indes im Praxisbeispiel, das den Betroffenen nur die Wahl zwischen einer breiten oder verweigerten Einwilligung lässt.

Für die praktische Handhabbarkeit eines solchen dynamischen, projektbezogenen Einwilligungsprozesses kann zunehmend auf die wachsenden technischen Möglichkeiten zurückgegriffen werden[61]. Digitale Plattformen, Patientenportale der Krankenhäuser oder die flächendeckend einzuführende elektronische Patientenakte können Betroffene dabei unterstützen, ihre Einwilligung je nach Forschungsfrage anzupassen, zu ändern oder projektbezogen zu widerrufen[62]. Die gewählte Einstellung könnte in einem Einwilligungsmanagement hinterlegt werden, das die Datennutzung entsprechend einem Berechtigungskonzept ermöglicht[63]. Digitale Plattformen können zudem als Kommunikationsschnittstelle des dynamischen Einwilligungsprozesses mit elektronischen Aufklärungsinstrumenten ergänzt werden[64]. Das charmante an diesen Lösungen ist, dass damit gleichzeitig der Anforderung an einen Datenschutz durch Technikgestaltung gem. Art. 25 DSGVO entsprochen werden kann. Die Wahl des Kommunikationskanals muss jedoch auch weniger technikaffine Menschen i. R. analoger, etwa schriftlicher oder mündlicher Einwilligungsprozesse berücksichtigen.

Das gegen einen dynamischen Ansatz vorgebrachte Argument, hierdurch werde die Forschung vor unverhältnismäßige Hürden gestellt[65], entkräftet nicht die datenschutzrechtliche Notwendigkeit einer in sachlicher und institutioneller Hinsicht informierten Entscheidung des Betroffenen. Ein unverhältnismäßiger Aufwand für das Forschungsvorhaben stellt nach geltendem Datenschutzrecht gerade kein Argument für das Abweichen von der konkreten Zweckfestlegung dar. Sowohl die DSGVO als auch die angepassten nationalen Datenschutzregeln sehen einen solchen forschungsbehindernden Aufwand stattdessen als Anwendungsfall der gesetzlichen Forschungsklauseln[66]. Art. 9 Abs. 2 lit. a DSGVO fordert dagegen durchweg einen festgelegten Zweck und lässt ein Abweichen hiervon nur zum Anheben der an die informierte Einwilligung gestellten Mindestanforderungen zu[67]. Sofern die mit der dynamischen Einwilligung verbundene Re-Kontaktierung des Betroffenen in der Praxis unerwünscht ist oder nicht umsetzbar erscheint, bleibt letztlich nur die Festlegung eines spezifischen Forschungszwecks durch einen bestimmten Verantwortlichen von Beginn an. Dabei dürfte ausreichend sein, wenn die Einwilligung für bestimmte, explizit genannte Krankheitsbilder erteilt wird, wie etwa die Krebsforschung[68].

#### 3. Einordnung des Praxisbeispiels

Vor diesem Regel-Ausnahme-Hintergrund erscheint es bedenklich, dass im Mustertext der MI-I die sachlich und

58) Für eine solche dynamische Einwilligung: *Cornelius*, MedR 2017, 15, 20; *Mausbach*, Jusletter 28.1.2019, Abstract; in diese Richtung *Molnár-Gábor*, in: *Bartfeld et al.*, Organoide, 2020, S. 237, 251; *Raum*, in: *Ehmann/Selmayr*, DSGVO, 2. Aufl. 2018, Art. 89, Rdnr. 36; *Schneider*, Sekundärnutzung klinischer Daten, 2015, S. 119–122; *Ulbricht/Weber*, in: *Friedewald et al.*, Informationelle Selbstbestimmung im digitalen Wandel, 2017, S. 265, 278; *Weichert*, Big Data im Gesundheitsbereich, ABlDA, 2018, S. 126; für den Einzelfall angepasste Einwilligungen, wie *dynamic consent*, auch Gutachten Datenethikkommission, 2019, S. 126, https://www.bmi.bund.de/SharedDocs/downloads/DE/publikationen/themen/it-digitalpolitik/gutachten-datenethikkommission.pdf?__blob=publicationFile&v=6 (8.2.2022); ähnlich *EDPS*, Opinion on scientific research, 2020, S. 20 („dynamic consent [is a] promising practice that should be further encouraged and developed“).

59) *Metschke/Wellbrock*, Datenschutz in Wissenschaft und Forschung, 2002, S. 27.

60) Da die Wahl zwischen breiter oder dynamischer Einwilligung den Betroffenen überlassen wird, schränkt die Steigerung der Einwilligungsanforderungen durch dynamische Einwilligung auch nicht die informationelle Selbstbestimmung des Betroffenen in rechtfertigungsbedürftiger Weise ein, anders *v. Kielmansegg*, in: TMF-Stellungnahme „Datenspende“, 2020, S. 97.

61) Sachverständigenrat, Gutachten Digitalisierung, 2021, S. 207; ähnlich *Augsberg/v. Ulmenstein*, GesR 2018, 341, 346.

62) Sachverständigenrat, Gutachten Digitalisierung, 2021, S. 207; vgl. auch *Cornelius*, MedR 2017, 15, 20 zu „First Genetic Trust“; ähnlich EDPS, Opinion on scientific research, 2020, S. 14 („via an IT interface“); *Martini/Hohmann*, NJW 2020, 3573, 3575; *Mausbach*, Jusletter 28.1.2019, Rdnrn. 4–6; *Ulbricht/Weber*, in: *Friedewald et al.*, Informationelle Selbstbestimmung im digitalen Wandel, 2017, S. 265, 278. Auch die *Datenethikkommission* empfiehlt die Entwicklung und Ausgestaltung von Privacy Management Tools (PMT), die dem Einzelnen die Einwilligungsverwaltung durch technische Lösungen erleichtert sowie Personal Information Management Systems (PIMS), die auf der Dienstleistungsebene für den Betroffenen nach dessen Wahl Entscheidungen übernehmen können, *Datenethikkommission*, Gutachten 2019, S. 126, 133.

63) *Weichert*, Big Data im Gesundheitsbereich, ABlDA, 2018, S. 124.

64) *Mausbach*, Jusletter 28.1.2019, Rdnr. 8; *Ulbricht/Weber*, in: *Friedewald et al.*, Informationelle Selbstbestimmung im digitalen Wandel, 2017, S. 265, 278.

65) *Koch*, Das Biobankgeheimnis, 2013, S. 192; *Mand*, MedR 2005, 565, 573; *Steinsbekk et al.*, Eur J Hum Genet 21 (2013), 897, 899; s. auch die Auffassung i. R. d. MI-I bei *Hänold*, ZD-Aktuell 2020, 06954.

66) Beispielhaft regelt § 13 Abs. 1 LDSG BW, der Art. 9 Abs. 2 lit. j DSGVO in Baden-Württemberg konkretisiert, dass öffentliche Stellen personenbezogene Daten für wissenschaftliche Forschungszwecke verarbeiten dürfen, wenn diese Zwecke auf andere Weise nicht oder nur mit unverhältnismäßigem Aufwand erreicht werden können und die Forschungsinteressen die Betroffeneninteressen überwiegen.

67) Art. 9 Abs. 2 lit. a 2. Halbs. und Abs. 4 DSGVO. Eine solche Verschärfung der Mindestanforderungen liegt etwa in vereinzelten mitgliedstaatlichen Schriftformerfordernissen (z.B. § 25 Abs. 3 LKHG Bln; § 4 Abs. 1 S. 1 GDSG NRW).

68) Vgl. *Spranger*, MedR 2017, 864.

institutionell weite Zweckfestlegung gerade nicht auf die initiale Datenspeicherung beschränkt ist oder davon abhängt, ob die Zweckfestlegung aufgrund des wissenschaftlich ergebnisoffenen Vorgehens auch im zeitlichen Verlauf ausgeschlossen bleibt. Vielmehr fußt die gesamte Forschungsstruktur der MI-I dauerhaft auf der initial eingeholten breiten Einwilligung. Eine Erneuerung oder sukzessive Konkretisierung der Einwilligung ist während der 30 Jahre der Datennutzung nicht vorgesehen. Hierdurch wird die eigentlich als Ausnahme gedachte breite Einwilligung (derzeit) zum Regelfall der Forschungsstruktur erklärt[69].

Dennoch erhielt der Mustertext der MI-I von der DSK 2020 ein zustimmendes Votum[70], was mit Blick auf die Stellungnahme der DSK aus 2019 beachtlich erscheint. Dagegen hatte sich der BfDI in seiner Stellungnahme zum Mustertext der MI-I noch für einen *dynamic* und gegen einen *broad consent* ausgesprochen[71].

Positiv in der neuesten Version des Mustertextes ist anzumerken, dass die Betroffenen nunmehr auf einer Internetseite einsehen können, welche Studien mit den Patientendaten durchgeführt werden. Auch können sie sich für einen E-Mail-Verteiler registrieren, um fortlaufend über neue Forschungsprojekte informiert zu werden[72]. Diese prozeduralen Informationsinstrumente sind zu begrüßen. Sie sorgen für Transparenz und Vertrauen bei den Betroffenen. Dennoch ersetzt die Information allein nicht die Einholung einer vorherigen, konkretisierten Zustimmung, sondern stellt bestenfalls eine dynamische Informationsvermittlung für einen möglichen aufgeklärten Opt-out dar.

Ebenfalls positiv anzumerken ist, dass die breite Einwilligung der MI-I eingebunden ist in eine Governance-Struktur, zu der weitere Sicherungsmaßnahmen, wie die Codierung (Pseudonymisierung) der Daten und das vorherige Votum der Ethik-Kommission gehören. Die institutionell nutzungsberechtigten Forschungsstellen, die außerhalb der datenerhebenden, behandelnden Einrichtung stehen, dürfen die klinischen Daten nur verschlüsselt und damit im Regelfall für sie faktisch anonym nutzen[73]. Dogmatisch passen diese kompensatorischen Schutzmaßnahmen indes weniger zur Rechtsgrundlage einer informierten Einwilligung. Vielmehr erinnern sie an die geeigneten Garantien zum Schutz der Betroffenenrechte als Element der gesetzlichen Rechtsgrundlage (Art. 9 Abs. 2 lit. j, 89 Abs. 1 DSGVO, national konkretisiert durch §§ 27 Abs. 1, 22 Abs. 2 BDSG)[74].

### D. Breite Einwilligung als Teil der gesetzlichen Forschungsklausel

Es wäre daher bedenkenswert, ob der von der MI-I präferierte Weg einer regelhaften breiten Einwilligung als Schutzgarantie i. R. datenschutzrechtlicher Forschungsklauseln fruchtbar gemacht werden könnte[75].

#### *I. Verhältnis Einwilligung und gesetzliche Forschungsklausel*

Zunächst ist festzuhalten, dass es zwischen der Einwilligung als Rechtsgrundlage und dem Rückgriff auf die gesetzliche Forschungsklausel nach geltendem Datenschutzrecht kein Vorrangverhältnis gibt. Teilweise wird zwar auch heute noch von einer Vorrangigkeit der informierten Einwilligung und der Subsidiarität der übrigen Rechtsgrundlagen ausgegangen. Dem wird hier nicht gefolgt. Die Rechtsgrundlagen nach Art. 6 Abs. 1 und Art. 9 Abs. 2 DSGVO stehen gleichrangig nebeneinander, worauf auch Art. 17 Abs. 1 lit. b DSGVO hinweist. Hiernach sind nach dem Widerruf der Betroffeneneinwilligung die Daten zu löschen, sofern nicht die Verarbeitung auf eine anderweitige Rechtsgrundlage gestützt werden kann. Dies verdeutlicht, dass Einwilligung und sonstige Erlaubnistatbestände als gleichrangige Rechtsgrundlagen nebeneinander stehen[76].

#### *II. Autonomiesichernde Elemente i. R. d. Forschungsklausel*

Art. 9 Abs. 2 lit. j DSGVO sieht spezifisch für die Forschung mit Gesundheitsdaten eine Öffnungsklausel für Rechtsgrundlagen vor, die gleichrangig neben die informierte Einwilligung treten. Die nationale Anpassung dieser Öffnungsklausel muss jedoch nicht zwangsläufig auf jegliche Form der Betroffenenbeteiligung verzichten[77]. Auf Bundesebene sieht § 27 Abs. 1 S. 1 BDSG eine Rechtsgrundlage für die Verarbeitung zu wissenschaftlichen Forschungszwecken ohne Einwilligung vor, wenn das verfolgte Forschungsinteresse das Betroffeneninteresse erheblich überwiegt[78]. Aus der privilegierten Datenverarbeitung zu Forschungszwecken „ohne Einwilligung" folgt ebenfalls nicht zwingend, dass auf autonomiesichernde Maßnahmen gänzlich verzichtet werden muss[79]. Da § 27 Abs. 1 BDSG eigenständig und gleichrangig neben die Einwilligung tritt, kann sowohl nach Sinn und Zweck als auch nach Wortlaut und Systematik „ohne Einwilligung" als „ohne informierte Einwilligung als Rechtsgrundlage" ausgelegt werden. Wie dargelegt, stellt die anfangs ausnahmsweise ausreichende breite Einwilligung nach Wegfall der Ausnahmeberechtigung gerade keine taugliche Rechtsgrundlage mehr dar. Sie kann aber durchaus als gegenüber der von Anfang an einwilligungsunabhängigen Datenverarbeitung als datenschutzfreundlichere Verarbeitungsvariante und Schutzgarantie i. S. v. Art. 89 Abs. 1 DSGVO und § 27 Abs. 1 S. 2 i. V. mit § 22 Abs. 2 S. 2 BDSG betrachtet werden, die zugleich i. R. d. Interessenabwägung zu berücksichtigen ist[80]. Hierzu passt, dass die anfänglich breite Einwilligung nur Teil einer hinzutretenden Schutzstruktur ist, die ergänzt wird durch

69) Allerdings plant die MI-I künftig auch ein dynamisches Einwilligungsmanagement zu ermöglichen, Sachverständigenrat, Gutachten Digitalisierung, 2021, S. 207.

70) DSK, Pressemitteilung v. 24. 4. 2020, https://www.datenschutzkonferenz-online.de/media/pm/20200427_Einwilligungsdokumente_der_Medizininformatik-Initiative.pdf, S. 1 (8. 2. 2022).

71) Vgl. *Hänold*, ZD-Aktuell 2020, 06954.

72) MI-I, Mustertext Patienteneinwilligung, V 1.6d, 2020, S. 3.

73) Zu personenbezogenen, pseudonymisierten und faktisch anonymen Daten im biomedizinischen Forschungskontext s. *Spitz/Cornelius*, in: *Richter et al.*, Datenreiche Medizin, 2021, 101 ff.

74) Zur Forschungsverarbeitung klinischer Daten ohne Einwilligung s. *Spitz et al.*, MedR 2021, 499, 502 ff.

75) Ähnlich der Vorschlag einer Zustimmungslösung als Garantie im Rahmen der Forschungsklausel beim *Sachverständigenrat*, Gutachten Digitalisierung, 2021, S. 232; *Shabani*, Mol Syst Biol 17 (2021), e10229, S. 1, 2 mit Verweis auf den Vorschlag der Kommission für ein Daten-Governance-Gesetz v. 25. 11. 2020, COM(2020) 767 final und den dort in Art. 2 Nr. 10 vorgesehenen „Datenaltruismus".

76) *Spitz et al.*, MedR 2021, 499, 503 m. w. N.

77) Vgl. *Spitz et al.*, MedR 2021, 499, 503 f.

78) § 27 Abs. 1 BDSG sowie verschiedene LDSG (z. B. § 13 Abs. 1 LDSG BW) gehen von einem weniger strengen Zweckbegriff („wissenschaftliche Forschungszwecke") als Art. 9 Abs. 2 lit. a DSGVO („festgelegte Zwecke") aus, wohingegen andere Landesregelungen „bestimmte Forschungsvorhaben" oder „bestimmte Forschungsprojekte" verlangen (im Krankenhauskontext, auch im Bereich der Einwilligung, z. B. § 39 Abs. 1, 2 LKHG Bremen; § 17 Abs. 1, 2 LKHG LSA und allgemein § 25 LDSG Bbg; § 9 LDSG M-V; § 13 LDSG Nds.; § 23 LDSG Saar). Da im vorliegenden Szenario die breite Einwilligung deshalb keine taugliche Rechtsgrundlage mehr bietet, weil der konkrete Forschungszweck nun benannt werden kann, ergibt sich hieraus jedoch keine Einschränkung für die Nutzung der jeweils anwendbaren Forschungsklausel. Dennoch wäre eine vereinheitlichende Regelung gerade für Verbundforschung wünschenswert, dazu sogleich im Text.

79) Für eine Widerspruchs- und eine Zustimmungslösung bereits *Spitz et al.*, MedR 2021, 499, 503 f.; vgl. auch *v. Kielmansegg*, in: Stellungnahme „Datenspende", 2020, S. 109 f.

80) In diese Richtung EDPS, Opinion on scientific research, 2020, S. 20.

die Einbindung der Ethik-Kommission, die ausschließlich codierte Forschungsverarbeitung und die transparente Information der Betroffenen via Webservices[81].

#### *III. Vorbilder für die breite Einwilligung als Teil der Forschungsklausel*

Als Vorbild für eine solche breite Einwilligung als Anwendungsfall der Forschungsklausel kann § 2d Abs. 3 FOG Österreich[82] herangezogen werden[83]. Die Regelung sieht ausdrücklich einen „broad consent" als Anpassung der Öffnungsklausel des Art. 9 Abs. 2 lit. j DSGVO vor. Im Umkehrschluss wird somit der *broad consent* nicht als Rechtsgrundlage nach Art. 6 Abs. 1 lit. a, 9 Abs. 2 lit. a DSGVO angesehen, sondern als Teil der Forschungsklausel[84].

Für die vorliegende Lösung spricht auch der Verweis des EDPB auf die Einwilligung im Rahmen der VO (EU) 536/2014[85]. Nach Erwägungsgrund 29 VO (EU) 536/2014 sollen Daten aus klinischen Studien zu Forschungszwecken für „Zwecke der medizinischen […] Forschung" auf Grundlage einer Einwilligung des Prüfungsteilnehmers gem. Art. 28 f. VO (EU) 536/2014 verwendet werden dürfen. Das EDPB betont, dass diese breite Einwilligungsform nicht die Einwilligungsvoraussetzungen der DSGVO ersetzen, jedoch eine zusätzliche verfahrensrechtliche Garantie im Rahmen der Forschungsklausel der DSGVO bilden könne[86].

#### *IV. Unterschied zur breiten Einwilligung als Rechtsgrundlage*

Eine breite Einwilligung als regelhafte Datenverarbeitungsgrundlage führt zu einer bedenklichen Verwässerung der wesentlichen Grundsätze informierter Einwilligung. Die in der Praxis zu findende Kumulation der breiten Einwilligung mit zusätzlichen Schutzmaßnahmen bewegt sich als Element der informierten Einwilligung im luftleeren Raum, da weder Art. 6 Abs. 1 noch Art. 9 Abs. 2 DSGVO gesteigerte, über die üblichen technischen und organisatorischen Maßnahmen hinausgehende Schutzgarantien je nach Informationsgrad innerhalb der Einwilligung vorsehen.

Anders ist die Situation i. R. d. Forschungsklauseln: Das Zusammenspiel der mit Schutzmaßnahmen kombinierten breiten Einwilligung lässt sich hier dogmatisch sauber in das Regelungsgeflecht einbinden. Hier sehen sowohl Art. 89 Abs. 1 DSGVO als auch § 22 Abs. 2 S. 2 BDSG zusätzliche Schutzgarantien vor. Dieses Vorgehen bietet für den Betroffenen einen gesteigerten Schutz. An die Stelle der wie selbstverständlich genutzten breiten Einwilligung, die unter Umständen Jahre zurückliegt, tritt eben jene breite Einwilligung als Bestandteil einer einzelfallbezogenen Interessenabwägung nach § 27 Abs. 1 S. 1 BDSG[87]. Diese kann jedoch aufgrund der anfänglichen breiten Einwilligung, kombiniert mit den genannten Schutzstrukturen, erleichtert zugunsten der Forschung ausfallen.

Zu berücksichtigen ist bei der hier vorgeschlagenen Lösung, dass die Einordnung der breiten Einwilligung als Element der Forschungsklausel zwingend mit einer über Art. 21 Abs. 6 DSGVO hinausgehenden Berücksichtigung des Betroffenenwiderspruchs i. R. d. Güterabwägung nach § 27 Abs. 1 S. 1 BDSG einhergehen muss[88]. Die vorliegende Einordnung der breiten Einwilligung als Teil der Forschungsklausel darf nicht dazu führen, dass der Betroffenenschutz, der in Gestalt der Widerrufsmöglichkeit (Art. 7 Abs. 3 DSGVO) bei Einordnung der breiten Einwilligung als informierte Einwilligung bestehen würde, nun durch das inadäquate Widerspruchsrecht aus Art. 21 Abs. 6 DSGVO unterschritten wird. Da der mit dem Widerspruch zum Ausdruck kommende entgegenstehende Betroffenenwille im Rahmen der gesetzlichen Forschungsklausel meistens dazu führt, dass die Güterabwägung zu Gunsten des Betroffeneninteresses ausfällt[89], bleibt die vorliegend vertretene Lösung nicht hinter dem Widerrufsrecht aus Art. 7 Abs. 3 S. 1 DSGVO zurück.

#### *V. Praktische Umsetzung*

Die Betroffenen sollten bereits bei der Aufklärung darüber informiert werden, dass zum aktuellen Zeitpunkt noch nicht alle künftigen Forschungsinhalte und nutzenden Forschungseinrichtungen genannt werden können, sodass ausnahmsweise eine breite Einwilligung eingeholt wird. Zugleich sollten die Betroffenen darauf hingewiesen werden, dass im zeitlichen Verlauf dynamisch über konkrete Forschungsvorhaben und Forschungsstellen informiert und ihre konkrete Einwilligung eingeholt wird, sobald dies möglich ist. Den Betroffenen könnte ermöglicht werden, auf diesen dynamischen Prozess als Ausdruck informationeller Selbstbestimmung zu verzichten[90]. Zusätzlich sollten die Betroffenen darüber informiert werden, dass unter Umständen die nachfolgende konkrete Forschungsverarbeitung auf die gesetzliche Forschungsklausel unter den dortigen Voraussetzungen gestützt werden kann (Art. 9 Abs. 2 lit. j DSGVO i. V. mit § 27 Abs. 1 BDSG). Es sollte erklärt werden, dass dies insbesondere dann geschieht, wenn die Betroffenen nicht erreichbar sind oder unüberwindliche praktische Hürden der erneuten Kontaktaufnahme entgegenstehen. Auf die Überprüfung dieser Voraussetzungen durch eine unabhängige Stelle (etwa eine

81) Zu einer den *broad consent* ergänzenden Governance-Struktur vgl. *Forum Gesundheitsforschung*, Strategiepapier Nutzbarmachung digitaler Daten, 2020, S. 29.

82) Bundesgesetz über allgemeine Angelegenheiten gemäß Art. 89 DSGVO und die Forschungsorganisation (Forschungsorganisationsgesetz – FOG), Österreichisches BGBl. 1981/341 i. d. F. BGBl. I 2018/31.

83) § 2d Abs. 3 FOG: „Im Anwendungsbereich dieses Bundesgesetzes ist die Verarbeitung von Daten (§ 2b Z. 5) gemäß Art. 9 Abs. 2 Buchstabe j DSGVO zulässig, wenn die betroffene Person freiwillig, in informierter Weise und unmissverständlich ihren Willen in Form einer Erklärung oder einer sonstigen eindeutigen bestätigenden Handlung bekundet, mit der Verarbeitung der sie betreffenden personenbezogenen Daten einverstanden zu sein, wobei die Angabe eines Zweckes durch die Angabe (1.) eines Forschungsbereiches oder (2.) mehrerer Forschungsbereiche oder (3.) von Forschungsprojekten oder (4.) von Teilen von Forschungsprojekten erfolgen darf (‚broad consent')."; hierzu eingehend *Haimberger*, Datenschutz in der medizinischen Forschung, 2021, S. 75 ff. m. w. N. zum österreichischen Umgang mit der Regelung.

84) *Haimberger*, Datenschutz in der medizinischen Forschung, 2021, S. 77; so auch die abstrakte DSFA (Art. 35 Abs. 10 und Erwägungsgrund 92 DSGVO) in Anh. 5 Datenschutz-Folgenabschätzung zu § 2d Abs. 3 FOG, S. 2.

85) Verordnung (EU) Nr. 536/2014 des Europäischen Parlaments und des Rates v. 16. 4. 2014 über klinische Prüfungen mit Humanarzneimitteln und zur Aufhebung der Richtlinie 2001/20/EG, Abl. EU L 158, S. 1.

86) EDPB, Leitlinien 5/2020 v. 4. 5. 2020, S. 36, Rdnr. 153.

87) Zu § 27 Abs. 1 S. 1 BDSG *Spitz et al.*, MedR 2021, 499, 502.

88) Zum (ungeschriebenen) Widerspruchsmodell als Teil der Güterabwägung des § 27 Abs. 1 S. 1 BDSG *Spitz et al.*, MedR 2021, 499, 503; zum Umgang mit Betroffenenwidersprüchen im österreichischen § 2d Abs. 3 FOG *Haimberger*, Datenschutz in der medizinischen Forschung, 2021, S. 83 ff.

89) Zur Berücksichtigung des Betroffenenwiderspruchs i. R. gesetzlicher Forschungsklauseln *Metschke/Wellbrock*, Datenschutz in Wissenschaft und Forschung, 2002, S. 12.

90) Eine dynamisierte Einwilligung ergänzt um weitere Anpassungsoptionen des Betroffenen als „Gold-Standard" bezeichnend *Augsberg/v. Ulmenstein*, GesR 2018, 341, 344. Modulare Abweichungen nach Wahl des Betroffenen werden auch unter dem Stichwort des *Meta consent* diskutiert, *Kaye et al.*, Eur J Hum Genet 23 (2015), 141, 143; *Ploug/Holm*, Bioethics 30 (2016), 721 ff.; *dies.*, BMC Med Ethics 18 (2017), 51.

Ethik-Kommission) und zusätzliche Schutzmaßnahmen wie die ausschließlich codierte Forschungsverarbeitung und die stets gegebene Widerspruchsmöglichkeit, sind die Betroffenen hinzuweisen.

#### E. Ergebnis

Der vom Mustertext der MI-I vorgezeichnete Weg scheint insoweit präjudiziert, als die DSK diesen als datenschutzkonform ansieht. Gleichwohl steht die hier zum Regelfall erhobene breite Einwilligung nach vorliegender Auffassung im Spannungsverhältnis mit dem datenschutzrechtlichen Ausnahmecharakter breiter Einwilligung. Insoweit erscheint zweifelhaft, ob das EDPB und/oder die Rechtsprechung diesen Weg mittragen würde. Dabei eröffnet wenigstens das europäische Datenschutzrecht Instrumente, um eine im Regelfall unverwässerte informierte Einwilligung im Forschungskontext zu erhalten und zugleich Forschung ausreichend zu privilegieren.

Dieser Weg sollte auch national beschritten werden, indem (i) der Regelfall ein festgelegter sachlicher und institutioneller Einwilligungszweck ist, von dem im Forschungskontext nur ausnahmsweise abgewichen werden darf, wenn das der Forschung inhärente inkrementelle Streben nach Erkenntnis eine genauere Zweckfestlegung verhindert. Außerdem sollte (ii) die zuvor nur breit eingeholte Einwilligung durch einen dynamischen Einwilligungsprozess ergänzt werden, sobald der Forschungsverlauf die genaue Festlegung zulässt. Schließlich könnten (iii) die gesetzlichen Forschungsklauseln i. V. mit der anfänglich breiten Einwilligung für den Fall genutzt werden, dass der dynamische Einwilligungsprozess das Forschungsvorhaben vor unüberwindbare Hürden stellt.

Eine solche Vorgehensweise sollte künftig durch eine gesetzliche Einbettung auf solide, bundeseinheitliche Füße gestellt werden. Die bestehende Regelung in § 2d Abs. 3 FOG Österreich deutet diesen legislativen Weg an. Dies sollte der Gesetzgeber zügig angehen, um den bestehenden Rechtsunsicherheiten zu begegnen. Insbesondere das „erhebliche Überwiegen" des Forschungsinteresses stößt in der praktischen Umsetzung auf Risiken. Es wäre insoweit Aufgabe des Gesetzgebers, für eine Entschärfung und Präzisierung der Forschungsklausel zu sorgen, etwa durch die Benennung von beispielhaften Fallgruppen im Rahmen des § 27 BDSG. Eine nationale Ergänzung der Forschungsklausel könnte zugleich die uneinheitliche Anwendbarkeit von § 27 Abs. 1 BDSG im Kontext von Krankenhäusern und Universitätskliniken konsolidieren. Dies wäre in ähnlicher Form vorstellbar, wie dies schon jetzt § 287a S. 1 SGB V[91] sektorspezifisch vorsieht. Insoweit bleibt zu hoffen, dass das im Koalitionsvertrag angedeutete Gesundheitsdatennutzungsgesetz zur besseren wissenschaftlichen Nutzung von Patientendaten künftig für mehr Klarheit bei der so wichtigen wissenschaftlichen Sekundärnutzung klinischer Daten in der Praxis sorgen wird.

**Open Access.** Dieser Artikel wird unter der Creative Commons Namensnennung 4.0 International Lizenz veröffentlicht, welche die Nutzung, Vervielfältigung, Bearbeitung, Verbreitung und Wiedergabe in jeglichem Medium und Format erlaubt, sofern Sie den/die ursprünglichen Autor(en) und die Quelle ordnungsgemäß nennen, einen Link zur Creative Commons Lizenz beifügen und angeben, ob Änderungen vorgenommen wurden.
Die in diesem Artikel enthaltenen Bilder und sonstiges Drittmaterial unterliegen ebenfalls der genannten Creative Commons Lizenz, sofern sich aus der Abbildungslegende nichts anderes ergibt. Sofern das betreffende Material nicht unter der genannten Creative Commons Lizenz steht und die betreffende Handlung nicht nach gesetzlichen Vorschriften erlaubt ist, ist für die oben aufgeführten Weiterverwendungen des Materials die Einwilligung des jeweiligen Rechteinhabers einzuholen.
Weitere Details zur Lizenz entnehmen Sie bitte der Lizenzinformation auf http://creativecommons.org/licenses/by/4.0/deed.de.

91) Bei § 287a SGB V ist problematisch, ob die Regelung nur innerhalb oder auch außerhalb des bereichsspezifischen Sozialdatenschutzrechts gilt, für letzteres BfDI, Stellungnahme zum Gesetz zum Schutz der Bevölkerung, 2020, S. 3.

## Ärztliche Kooperation im Lichte des MoPeG

**Carsten Hansen**

#### Abstract

*Das Gesetz zur Modernisierung des Personengesellschaftsrechts (Personengesellschaftsrechtsmodernisierungsgesetz – „MoPeG") reformiert das Recht der Gesellschaft bürgerlichen Rechts („GbR"), eröffnet aber mit der Öffnung der Personenhandelsgesellschaften für Freiberufler auch neue Formen der Kooperationen von Ärztinnen und Ärzten*[1].

#### I. Einleitung

Das MoPeG wurde am 17. 8. 2021 im Bundesgesetzblatt verkündet[2]. Bis zum Inkrafttreten des überwiegenden Teils der Regelungen des MoPeG am 1. 1. 2024[3] besteht für die als GbR organisierte Zusammenarbeit von Ärzten Prüfungsbedarf, ob die Gesellschaftsverträge noch zu dem dann geltenden Regelwerk, über das im Folgenden ein Überblick gegeben wird, passen. Darüber hinaus ist bei Kooperationen von Ärzten generell zu prüfen, welche Kooperationsform – auch im Hinblick auf die Möglichkeit zur Wahl einer oHG oder (GmbH & Co.) KG – zukünftig (noch) die Richtige ist.

#### II. Formen der ärztlichen Kooperation

Die häufigsten Formen ärztlicher Kooperation, auf die man in der Praxis trifft, sind die Berufsausübungsgemeinschaft,

Rechtsanwalt Dr. iur. Carsten Hansen LL.M. (NZ),
Wachtberg, Deutschland

1) In dem folgenden Beitrag wird aus Gründen der besseren Lesbarkeit ausschließlich die männliche Form verwendet. Sie bezieht sich auf Personen beiderlei Geschlechts sowie Diverse.
2) BGBl. I S. 3436.
3) Vgl. Art. 137 S. 1 MoPeG.

Funding

Open access funding enabled and organized by Projekt DEAL.

